# Current practice and perspectives in CRO oversight based on a survey performed among members of the German Association of Research-Based Pharmaceutical Companies (vfa)

**DOI:** 10.3205/000243

**Published:** 2017-01-26

**Authors:** Michael Hennig, Ferdinand Hundt, Susanne Busta, Stefan Mikus, Per-Holger Sanden, Andrea Sörgel, Thorsten Ruppert

**Affiliations:** 1 GlaxoSmithKline GmbH & Co. KG, Munich, Germany; 2 Doc-Dog-Consulting, Berlin, Germany; 3 Bristol-Myers Squibb, Munich, Germany; 4 Pfizer Pharma GmbH, Berlin, Germany; 5 Merck KGaA, Darmstadt, Germany; 6 MSD Sharp & Dohme GmbH, Munich, Germany; 7 vfa, Berlin, Germany

**Keywords:** clinical trial, outsourcing, CRO, vendor, oversight, supervision, quality management

## Abstract

In recent years, the number and scope of outsourced activities in the pharmaceutical industry have increased heavily. In addition, also the type of outsourcing has changed significantly in that time.

This raises the question of whether and how sponsors retain the capability to select and to control the contract research organizations (CROs) involved and what expertise still has to be present in the development department as well as other relevant departments to ensure adequate oversight, also in line with the expectations of regulators and health authorities. In order to answer these questions, a survey was conducted among the German vfa member companies. The survey describes the latest developments and experiences in outsourcing by 18 German vfa member companies. It concentrates on measures how to implement Quality Assurance (QA) when performing outsourced clinical studies.

This study shows that the majority of companies apply a full-outsourcing, preferred-provider model of clinical trial services, with the clinical research department playing the major role in this process. A large amount of guiding documents, processes and tools are used to ensure an adequate oversight of the services performed by the CRO(s).

Finally the guiding principles for all oversight processes should be transparent communication, a clearly established expectation for quality, a precise definition of accountability and responsibility while avoiding silo mentality, and a comprehensive documentation of the oversight’s evidence. For globally acting and outsourcing sponsors, oversight processes need to be aligned with regards to local and global perspectives.

This survey shows that the current implementation of oversight processes in the participating companies covers all relevant areas to ensure highest quality and integrity of the data produced by the outsourced clinical trial.

## Zusammenfassung

In den letzten Jahren haben sich sowohl die Anzahl als auch der Umfang der ausgelagerten Tätigkeiten in der pharmazeutischen Industrie stark erhöht. Darüber hinaus hat sich auch die Art des Outsourcings in dieser Zeit verändert.

Dies wirft die Frage auf, ob und wie die Sponsoren die Fähigkeit, geeignete CROs auszuwählen und zu überprüfen, beibehalten und welches Know-how noch in den Forschungs-, Entwicklungs- und anderen Abteilungen vorhanden sein muss, um eine angemessene Aufsicht zu gewährleisten, wie sie auch vom Gesetzgeber und den Überwachungsbehörden erwartet wird. Um diese Fragen zu beantworten, wurde eine Umfrage unter den deutschen vfa-Mitgliedsunternehmen durchgeführt. Die Studie beschreibt die neuesten Entwicklungen und Erfahrungen im Outsourcing von 18 deutschen Vfa-Mitgliedsunternehmen. Es werden Maßnahmen zur Umsetzung von Quality Assurance (QA) beschrieben, die bei der Auslagerung von klinischen Studien angewendet werden sollten.

Diese Studie zeigt, dass die Mehrheit der Unternehmen ein Full-Outsourcing-Modell mit bevorzugten Anbietern („Preferred Provider“) im Bereich der klinischen Studien anwendet; dabei spielt die Abteilung „Klinische Forschung“ die Hauptrolle in diesem Prozess. Eine große Menge von Guidelines, Prozessen und Werkzeugen werden verwendet, um eine angemessene Aufsicht über die Leistungen von den CROs zu gewährleisten.

Schließlich sollten die Leitprinzipien für alle Oversight-Prozesse folgende Punkte umfassen: transparente Kommunikation, klar festgelegte Erwartung hinsichtlich der Qualität, genaue Definition der Verantwortlichkeiten ohne Silodenken, sowie eine umfassende Dokumentation der Oversight-Tätigkeiten. Für global agierende Sponsoren sollten die Oversight-Prozesse in Bezug auf lokale und globale Perspektiven abgestimmt sein.

Diese Studie zeigt, dass die aktuelle Implementierung der Oversight-Prozesse in den beteiligten Unternehmen alle relevanten Bereiche abdeckt, um höchste Qualität und Integrität der durch die ausgelagerten klinischen Studien generierten Daten zu gewährleisten.

## 1 Introduction

In recent years, the number and scope of outsourced activities in the pharmaceutical industry have increased heavily. In addition, also the type of outsourcing has changed significantly in that time. 

In the past the majority of clinical study activities were performed largely in-house. Most activities, especially regarding Quality Management (QMS – Quality Management System and CAPA – Corrective Actions/Preventive Actions) were done by the sponsor itself, and only individual activities were awarded to specialized contract research organizations (CROs). Today the trend is increasingly towards completely outsourced studies with a full-service provider and a so-called strategic partnership between a sponsor and its main CRO (preferred provider). Major areas of previous sponsor tasks are assumed by CROs, including Quality Management – however, according to ICH E6, “*the ultimate responsibility for the quality and integrity of the trial data always resides with the sponsor*” [1] and in the recent update (R2) of the guideline the amended introduction describes the objective "*to encourage implementation of improved and more efficient approaches to *[...]* oversight *[...]" [2]. Already before, the health authorities made clear in their last year's inspections that it is the sponsor’s responsibility to actively ensure by oversight that trial conduct follows Good Clinical Practice (GCP). Consequently e.g. the FDA (U.S. Food and Drug Administration) issued more and more 483s (list of inspectional observations) and warning letters directly to the sponsor that in the past were mainly issued to investigators and CROs.

This trend towards outsourcing is illustrated by 375 industry professionals who responded to Contract Pharma’s Eleventh Annual Outsourcing Survey 2015 [3]. Forty-five percent of respondents were from pharmaceutical sponsor companies, and the remaining 55% represented service providers. When asked if there is an increasing demand for outsourcing, 80% of respondents answered yes. The number one reason for this, according to 41% of respondents, is to focus on core competencies. Pharmaceutical company sponsors say they are also outsourcing more because they are virtual (30%), while 14% say they lack the capabilities in-house. Companies were focusing their outsourcing efforts in 2015 on the following fields: analytical and testing services (37%); clinical trials, phases I-IV (34%); API (active pharmaceutical ingredient) manufacturing (31%); solid dosage manufacturing (28%); formulation development (20%); clinical trials materials (15%). 

These figures demonstrate the high relevance of outsourcing in clinical trials run by the pharmaceutical industry.

As a result, the competent authorities like EMA (European Medicine Agency) and FDA increased their expectations of oversight of service providers by the sponsor and focus on this aspect during inspections. Important to note is that competent authorities do not limit the need for quality management to specific activities like monitoring but e.g. “*FDA considers monitoring to be just one component of a multi-factor approach to ensuring the quality of clinical investigations*” [4]. The EMA as well proposes a risk-based approach to quality management including oversight in their reflection paper [5]. In consequence, proof of a broader scope of oversight is demanded in inspections.

Furthermore, in the recent Addendum of the ICH-GCP a new sentence has been added in 5.2. regarding the involvement of a CRO stating that „*the sponsor should ensure oversight of any trial-related duties and functions carried out on its behalf.*” [2]. All this raises the question of whether and how sponsors retain the capability to select and to control the CRO(s) involved and what expertise still has to be present in the development department as well as other relevant departments to ensure adequate oversight. In order to answer these questions, a survey was conducted among the German Association of Research-Based Pharmaceutical Companies (vfa; Verband forschender Arzneimittelhersteller) member companies. The survey results first describe the latest developments and experiences in outsourcing by the German vfa member companies and second concentrates on measures how to implement Quality Assurance (QA) when performing outsourced clinical studies.

## 2 Methods

The vfa, the Association of Research-Based Pharmaceutical Companies, is the trade organization of research-based pharmaceutical companies in Germany which represents 2/3 of the pharmaceutical market in Germany. 44 leading research-based pharmaceutical companies are currently organized in the vfa. In a joint project of the sub-committee on clinical trials and quality assurance (UA KliFo/QS) and the working group Biostatistics within the vfa, a questionnaire (see Attachment 1) covering the major aspects on the current practice of CRO selection and oversight was developed – in these committees 25 vfa member companies are involved. This survey was based on a first version of a questionnaire covering mainly biostatistics and data management aspects of CRO oversight, developed in 2014 by the working group Biostatistics of the vfa. 

The questionnaire referred to interventional clinical studies of phases II–IV, as studies of these phases are similar with regards to the outsourced services. It started with a section, in which the key elements were defined – to ensure a common understanding and interpretation of these elements, as shown below:

- The term “CRO oversight” is used for any measure to control the performance, the deliverables and the efficiency of contract research organizations (CROs) performing outsourced tasks on behalf of the pharmaceutical company or acting as the sponsor of a clinical study – not covered in this questionnaire: insourcing/temporary employment. Other terms typically used in this context include “CRO management”, “CRO supervision”.
- The term “preferred provider” is used for any outsourcing model, in which one or several CROs are selected as primary supplier by a pharmaceutical company in order to perform defined tasks for a series of clinical studies. Other terms typically used in this context include “strategic (alliance) partner/vendor/CRO”.
- The terms “local” and “global” refer to international companies with local subsidiaries in various countries. Here “global” refers to the CRO outsourcing on the international level within a company, whereas “local” refers to the German subsidiary and studies on the local German level – if applicable.

The questionnaire (see Attachment 1) consisted of three sections:

- In a *general part* questions about outsourcing models, the outsourced services, the selection and decision-making were asked. Here it was e.g. assessed whether the outsourcing is organized locally or globally as well as the reasons for outsourcing. The global and local perspectives were addressed separately as the vfa member companies are acting with a global and local focus.
- The second section dealt with the *procedures ensuring CRO oversight* and covered issues like CRO qualification, audits, SOPs, other oversight tools and escalation processes.
- The third part covered specific oversight topics for *the outsourcing of data management and biostatistics services*, e. g. requirements for data quality or coding. The results of this part will be published separately.

Finally the complete questionnaire covered 52 items. The survey was conducted from August till October 2015 and captured the companies’ outsourcing status quo applicable at this point in time. English language was selected for this questionnaire in order to ease the use within the companies. The questionnaire was sent out electronically by the vfa. The completed questionnaire was returned to the vfa and blinded afterwards by the vfa, ensuring that no identification of the companies was possible for the analysis team, lead by one of the authors (MH). Before analyzing the questionnaire descriptively, several quality control measures were performed in order to clean any data deficiencies and inconsistencies. In case of obvious data errors (e.g. an initial question was not answered, but the follow-up question was answered) the corresponding missing data was substituted. Some free text answers were clustered post-hoc by one of the authors (AS) to allow for a descriptive analysis of relevant categories. 

In addition, relevant articles were identified in a systematic literature search in Embase, Medline, and other internet sources, resulting in a total of 257 publications of potential relevance. After screening of the abstracts and full-texts finally a total of 10 relevant articles were selected [6], [7], [8], [9], [10], [11], [12], [13], [14], [15].

There are, according to our research, only a few articles concentrating on the quality aspect of CRO oversight [14], [16] in the field of clinical trials, most papers – not from peer reviewed journals – concentrated on operational aspects [7], [10], [11], [17], [18], [19] or extrapolated experiences with contract manufacturing organisations (CMOs) [6].

## 3 Results

Twenty-five companies within the vfa were contacted, from which 18 companies participated (72%). Three companies provided multiple feedbacks: One company provided two questionnaires – one covering the local (German) outsourcing perspective and one covering the global outsourcing perspective. One company provided three questionnaires: one covering the local perspective, one for the global perspective and one additional questionnaire covering the outsourcing of monitoring activities only. Finally one company divided their answers on two questionnaires: one for partly outsourcing activities, the other for full outsourcing activities.

In total the survey is based on 22 questionnaires from 18 different companies.

### 3.1 General questions

The first block of questions dealt with the general perspective of the outsourcing model.

#### Outsourcing models

All companies performed outsourcing of services to CROs. The majority of questionnaires (55%) referred to CRO outsourcing on an international/global level and to a full outsourcing model, in which all or the vast majority of services are outsourced (Table 1 (Tab. 1)). 

#### Full outsourcing

For those companies applying a full outsourcing model the vast majority (93%) used a preferred provider model. Within these models in 64% there was cooperation with more than one CRO acting as preferred provider, with an average of about 3 CROs per sponsor (Table 2 (Tab. 2)). 

#### Partly outsourcing

Companies applying a partly outsourcing model used CROs (90%) and freelancers (70%) as partners. For those companies performing a partly outsourcing model to CROs the selected CRO(s) typically acted as preferred provider (89%). In this model the majority of companies (63%) cooperated with one CRO as preferred provider (Table 3 (Tab. 3)).

**Figure 1:**
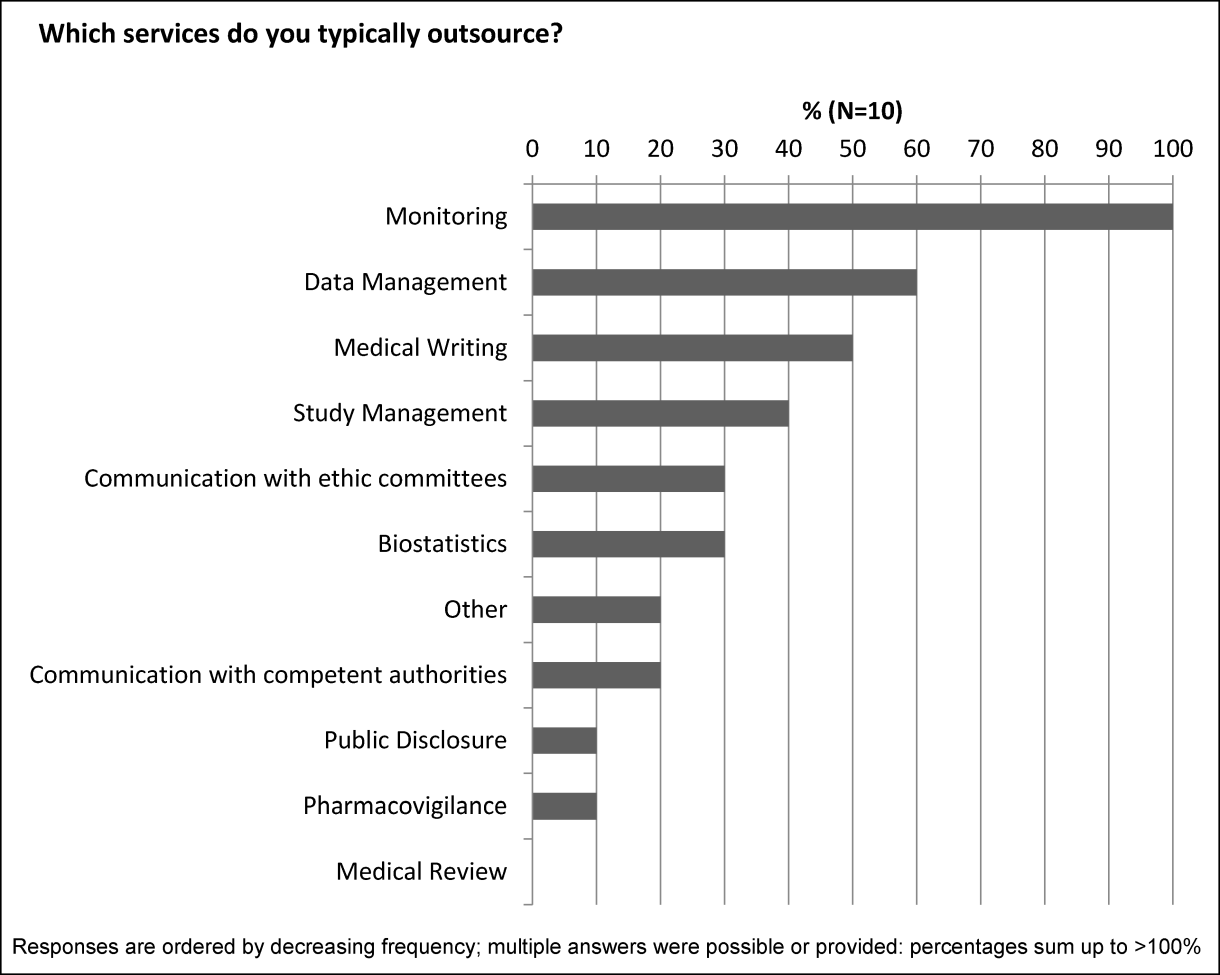
Partly outsourcing: Outsourced services

Monitoring services were outsourced by all companies performing a partly outsourcing model, followed by data management (60%) and medical writing services (50%) (Figure 1 (Fig. 1)).

#### Decision for outsourcing

A large number of sponsor departments were involved in the decision on which outsourcing model to apply for a specific study: the clinical research department is involved in 62%, followed by R&D business operations (38%), medical management (33%) and biostatistics (19%). The three main criteria for outsourcing services were “Decision by global” (73%), “Costs” (67%) and “Availability of internal resources/Flexibility in headcount planning” (60%). The main criterion for selecting specific services for outsourcing was “Decision by global/Strategic decision” (84%) (Table 4 (Tab. 4)).

#### Selection of preferred providers

When selecting a preferred provider the three key sponsor departments involved are: 1. Procurement, 2. Quality Management, and 3. Clinical Research Department (Table 5 (Tab. 5)).

#### Sponsor department involvement: Comparison of preferred provider/non-preferred provider model

**Figure 2:**
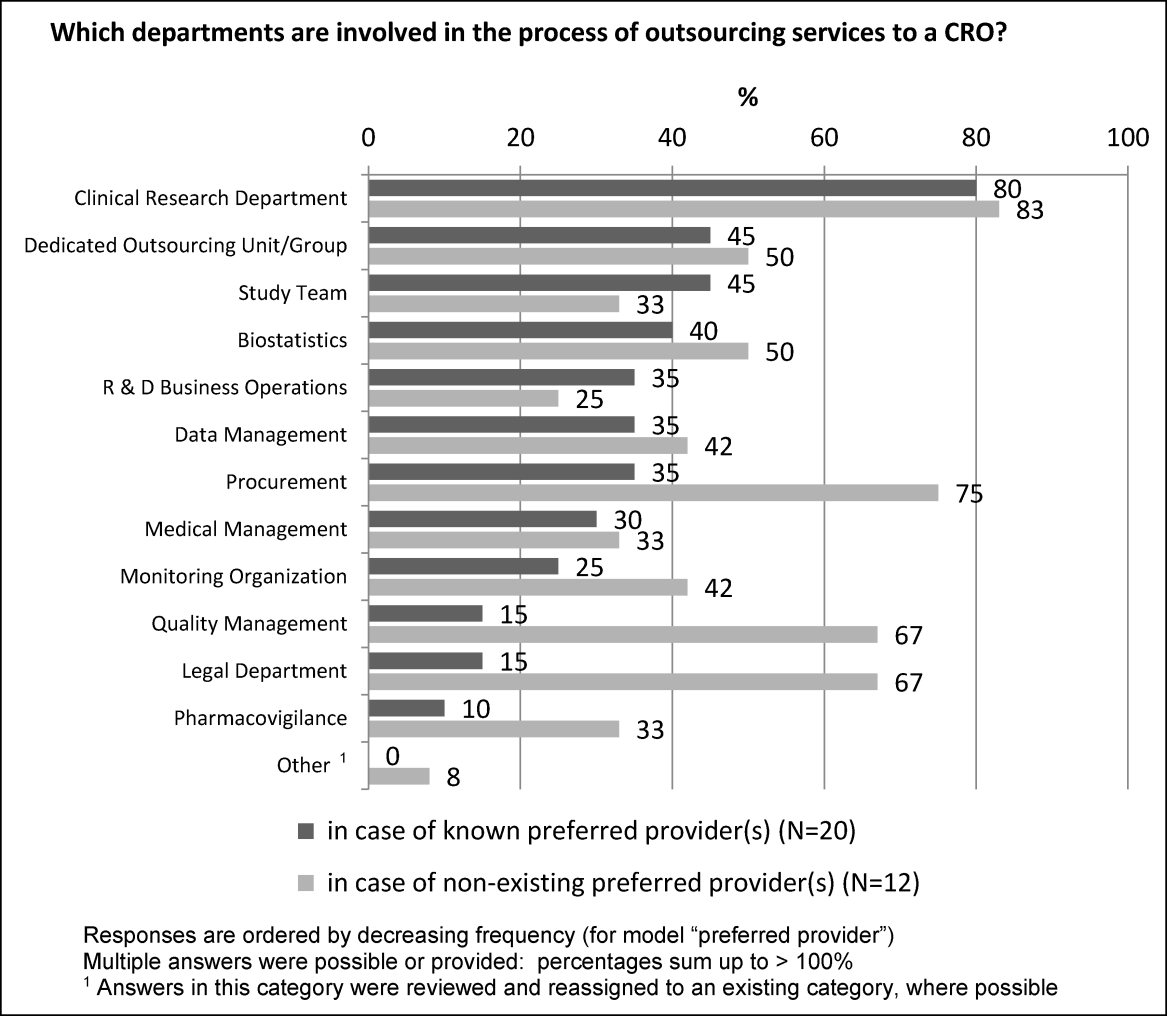
Departments involved in outsourcing

The involvement of sponsor departments into the process of outsourcing of services to a CRO was investigated for the two outsourcing models: a) preferred provider model and b) non-preferred provider model. In both models the clinical research department is the key department. The involvement of other departments like “Dedicated Outsourcing Unit”, “Study Team”, “Biostatistics”, “R&D Business Operations”, “Data Management”, “Medical Management” and “Monitoring Organization” was also similar for the two models. In the non-preferred provider model there was a more prominent involvement of the departments “Procurement”, “Quality Management”, “Legal Department” and “Pharmacovigilance” – compared to the preferred provider model (Figure 2 (Fig. 2)).

### 3.2 CRO oversight

#### Guiding documents

There is a SOP or any other quality/guiding document available in 81% of the responders. The following guiding documents are used: “Oversight plan”, “Partnership trainings“, „Guidance document on distribution of tasks”, “Job aides”. The vast majority (94%) of SOPs resp. guidance documents were declared as global documents (Table 6 (Tab. 6)).

#### Check of the CRO’s qualification before procurement 

The top-5-criteria checked during the CRO selection phase were: former experience with this CRO, costs, qualification of staff, and experience in indication, financial stability. These criteria were typically assessed by standardized documents like bid grids/templates (81%) or questionnaires (69%). A qualification audit prior to start of the study is performed in 69% of the responders (Table 7 (Tab. 7)). 

#### Extent and effort of CRO oversight during the study

A vendor audit is performed at least sometimes during the study in all responders; in 21% a vendor audit is a mandatory oversight tool. Those 79% of responders performing a vendor audit occasionally triggered an audit mainly by quality issues. The study duration was another trigger factor for an audit (39%).

Most of the responders (84%) used standardized tools for performing CRO oversight, like standardized metrics, meetings, oversight plans, action logs, monitoring visit cycle time reports, and regular CRO assessments.

A training program on how to perform CRO oversight is available in 76% of the responders. Also a risk-based CRO oversight based on the basis of previous experiences is conducted by 75%.

CRO oversight is typically conducted by the clinical research department (70%), quality management (65%) and the study team (60%).

A CRO oversight per CRO – across studies – (in the sense of an overall assessment) is performed by 85%.

A lessons learned process is implemented as mandatory requirement in 75% of the responders.

The documentation of CRO oversight measures is mostly done via meeting minutes (89%) and standardized documents (74%), like standardized oversight/surveillance plans, performance reports and metrics, action item logs and metrics/KPIs (key performance indicators). 

For implementation and support of the CRO oversights the following main instruments were used: RACI matrix (RACI: **R**esponsible, **A**ccountable, **C**onsulted and** I**nformed – a responsibility assignment matrix) of responsibilities (95%), matrix of valid SOPs (sponsor’s/CRO’s/both – as tick box) (86%), communication plan (86%) (Table 8 (Tab. 8)).

#### Size of CRO

**Figure 3:**
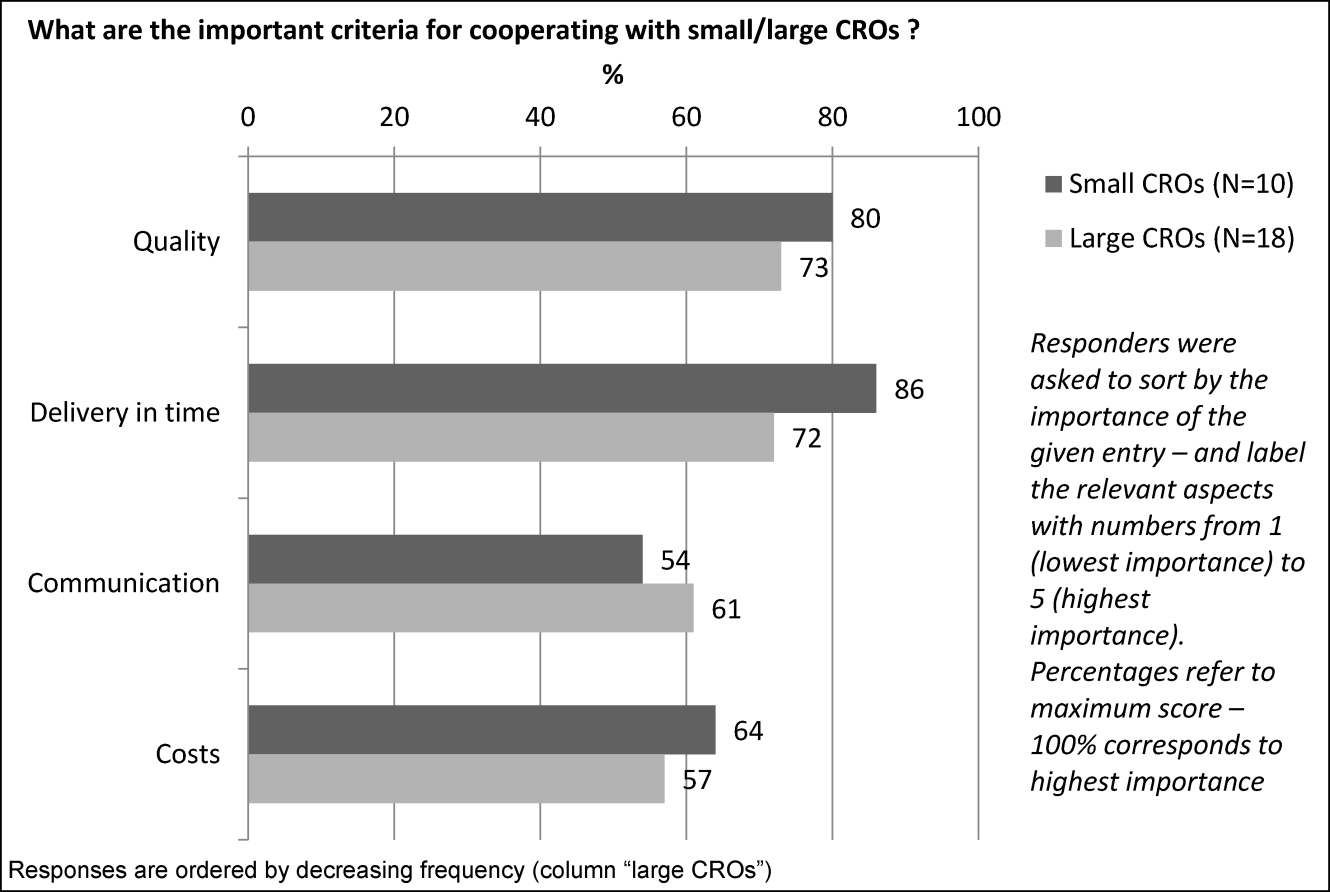
Criteria for small/large CROs

There was experience with large CROs in 18 responders (82%), compared to less experience with small CROs (10 responders, corresponding to 45%). The sequence of the main criteria for a partnership with a large CRO (ranked by importance) was: 1. Quality, 2. Delivery in time, 3. Communication, 4. Costs – compared to the following sequence for small CROs: 1. Delivery in time, 2. Quality, 3. Costs, 4. Communication (Figure 3 (Fig. 3)). 

The majority of responders (61%) have specific outsourcing areas dependent on the size of the CRO. Large CROs are typically selected for services like monitoring (67%), study management (56%) and data management (44%); whereas small CROs are typically selected for “other services” (60%) (Table 9 (Tab. 9)).

#### Multiple CROs

A minority of responders cooperate with several CROs for one study (29%). For them the main reason is the functional service provider strategy, and they use mainly portals and sharepoints for their exchange with the CROs (Table 10 (Tab. 10)).

#### Escalation

An established escalation plan is available in 90% of the responders. In case of QA findings all responders take action, with a timeframe depending on the issue (Table 11 (Tab. 11)).

## 4 Discussion

This study describes the implementation of CRO oversight measures in 18 Germany based pharmaceutical companies organized within the vfa. It shows that the majority of companies apply a full outsourcing, preferred provider model of clinical trial services, with the clinical research department playing the major role in this process. A large amount of guiding documents, processes and tools are used to ensure an adequate oversight of the services performed by the CRO(s).

This survey represents a large proportion of the current practice in pharmaceutical companies located in Germany, but it has to be taken into account that the representativeness is limited by three factors: the selection process of this survey, in which only 25 pharmaceutical companies represented in the vfa were considered, the return rate (of 72%) and by the fact that 3 companies submitted multiple questionnaires. 

Another limitation of this study is some degree of inconsistency in some answers, i.e. not all relevant questions were answered by all parties, leading to some missing values. This limitation however, is mainly caused by the diversity of the oversight process and also the oversight language within the participating companies.

The survey showed that the main criteria for selecting outsourcing services are “decisions by global” resp. “strategic decisions”, followed by “lack of internal resources”. This finding may be interpreted in the sense that the specific strategic reasons, typically considered on a global company level, are not always fully transparent to the company’s local affiliate executing the outsourcing measures. It seems that local experience and expertise with local CROs is only considered partly when deciding for an outsourcing strategy. Compared to a global perspective local affiliates may have more insight in CRO performance as they are actively overseeing the quality of CRO performance. It can be assumed that the remaining know-how within the sponsor is a critical issue for most of the companies, especially for small companies.

With regards to the size of a CRO, it turned out that small CROs are mostly preferred for services other than the “typical” services, as monitoring, study management, data management, etc. Although no further information on the kind of “other services” was collected, this may be interpreted as a niche for small CROs specialized on specific services like quality control visits, administrative services, recruitment services and laboratory services. It also seems that small CROs are preferred for services, where delivery in time is essential; whereas large CROs are preferred mainly because of the better communication. 

Although the large majority of participating companies report the existence of a SOP or any other guiding document, there remain five feedbacks from companies without these measures. This feedback may come from companies with only limited outsourcing-activities or from small companies, which have just started to set up a corresponding SOP-system.

The word “oversight” can be interpreted twofold: in the sense of supervision but also in the sense of error. Throughout this manuscript CRO oversight is used in the context of supervision, control, and project progress and quality.

The term “oversight” is also strongly related to the term “risk-based quality management”, topic of publications from authorities like the FDA (US) and the EMA (EU) but also from organizations like the ICH – International Council of Harmonisation [4], [5], [20], [21]. The FDA states in her procedural paper in 2013: “*Although sponsors can transfer responsibilities for monitoring to a CRO(s), they retain responsibility for oversight of the work completed by the CRO(s) that assume this responsibility. Sponsors should evaluate CRO compliance with regulatory requirements and contractual obligations in an ongoing manner. For example, sponsor oversight of monitoring performed by a CRO may include the sponsor’s periodic review of monitoring reports and vendor performance or quality metrics and documented communication between the sponsor and CRO regarding monitoring progress and findings.*” [4] 

This is in line with ICH E6, stating that *“the ultimate responsibility for the quality and integrity of the trial data always resides with the sponsor*” [1]. 

Looking into the EU, the “Reflection paper on risk based quality management in clinical trials” [5] concentrates on *the obligations of sponsors and/or CRO or vendors* to whom the sponsor has delegated trial related duties, risk assessment, risk control (risk mitigation/risk acceptance), quality tolerance limits concerning trial data, trial protocol procedures and trial management and risk review and reporting quality. Checking further the recommendations for risk-based quality management like ICH Q9, unfortunately nothing is mentioned concerning the sponsor-CRO relation [20].

There are some publications concerning quality management while outsourcing clinical trials [14], [16] focusing on “precontract audits” and on a four-phase program (credit history, qualification audit, audit during the conduct of the study, evaluation during and after by the department concerned).

In consequence various SOPs have been implemented by sponsors outsourcing clinical trials partly or completely. Internationally acting pharmaceutical companies organized in the vfa accomplished this with global guiding documents on e.g. vendor identification, vendor management and vendor qualification. Other guiding documents describe the RACI system (RACI: R=responsible, A=accountable, C=consulted, I=informed) defining the collaboration between sponsor and vendor in detail: i.e. start up, protocol development, site selection, monitoring, safety, and also project oversight/management). In some companies the entire process of vendor engagement and outsourcing activities have been centralized by creating a single platform/outsourcing department coordinating all related outsourcing tasks. In this model a standardized oversight process covering the vendor selection, the execution of oversight measures and the close-out activities has been established. 

With regards to the commonly used preferred provider model, some companies follow the approach to govern all interfaces between sponsor and preferred provider in one document, allowing both parties to act according to their SOPs. Other sponsors have established regular partnership meetings with the preferred CRO(s), in which a standardized regional review of all outsourced studies is performed. However, it seems that the oversight with non-preferred providers is less regulated by most sponsors, leaving more room for interpretation, which may result in lower quality.

There is definitely a very large number of tools available for performing and measuring the oversight – for all relevant levels, like: investigator level, study level, asset level, process level, enterprise level and relationship level. These tools cover aspects like on-site oversight visits, investigator site audits, study level performance metrics, study team meetings, quality standards, training events, balanced scorecards, etc. A single tool may be highly effective in one trial but only of limited value in another trial – this may also explain why none of the tools addressed in this survey reached a 100% consent. In order to establish an efficient CRO oversight it is rather essential to combine the relevant tools into a bundle. Here the key task for the sponsor is to identify and implement the adequate bundle of tools into an oversight plan for a specific study. 

In addition there are typically further local guiding documents like manuals and/or SOPs, regulating the local specifics within a country. The impact of all guiding documents should be checked on a regular basis e.g. by implementation of a lessons learned process as a mandatory requirement. This important element is established in the vast majority of companies participating in this survey.

As a consequence this leads to a large amount of SOPs, guidance documents, forms, templates – a total number of more than 20 relevant documents can be available. In this context it is of importance to highlight the relevant publication by Schmidt et al. to avoid overregulation, creating too much – unnecessary – interfaces, just endangering the original aim: quality [22]. As almost all registration studies are multinational, SOPs need to be globally usable. Therefore, global SOPs should describe all globally defined processes to ensure harmonization and efficiency across the whole organization. However, regional or local amendments to global SOPs need to be possible but should only be introduced if required by regional/local law/regulations or organizational structures of affiliates. A SOP should describe the standard situation of a process. Therefore, special rules and exceptions should be avoided. SOPs do not need to consider all imaginable situations. SOPs and associated workflows should be kept as simple as possible [22]. However, in relation to the guidance documents by EU and US authorities containing information on CRO oversight it seems required for sponsors, who outsource parts of or complete clinical trials, to implement quality management plans. These plans should contain information on how continuous CRO oversight on a global and local level is organized, including risk assessment, risk control and risk review (cycle) [5], including defined escalation processes. In this context involved functions should not treat their part of a study as an isolated piece of work because an integrated cross departmental and risk-based sponsor oversight approach can help to further increase the quality [23].

Finally the guiding principles for all oversight processes should be transparent communication, a clearly established expectation for quality, a precise definition of accountability and responsibility and a comprehensive documentation of the oversight’s evidence. For globally acting and outsourcing sponsors oversight processes need to be aligned with regards to local and global perspectives. This survey shows that the current implementation of oversight processes in the participating companies covers all relevant areas to ensure highest quality and integrity of the data produced by the outsourced clinical trial.

## 5 Conclusion

This survey shows that the current implementation of oversight processes in the participating companies covers all relevant areas to ensure highest quality and integrity of the data produced by the outsourced clinical trial. It remains the ultimate responsibility of any sponsor to apply the implemented measures adequately.

## Notes

### Competing interests

The authors declare that they have no competing interests.

## Supplementary Material

Questionnaire on current practice in CRO oversight at vfa companies

## Figures and Tables

**Table 1:**
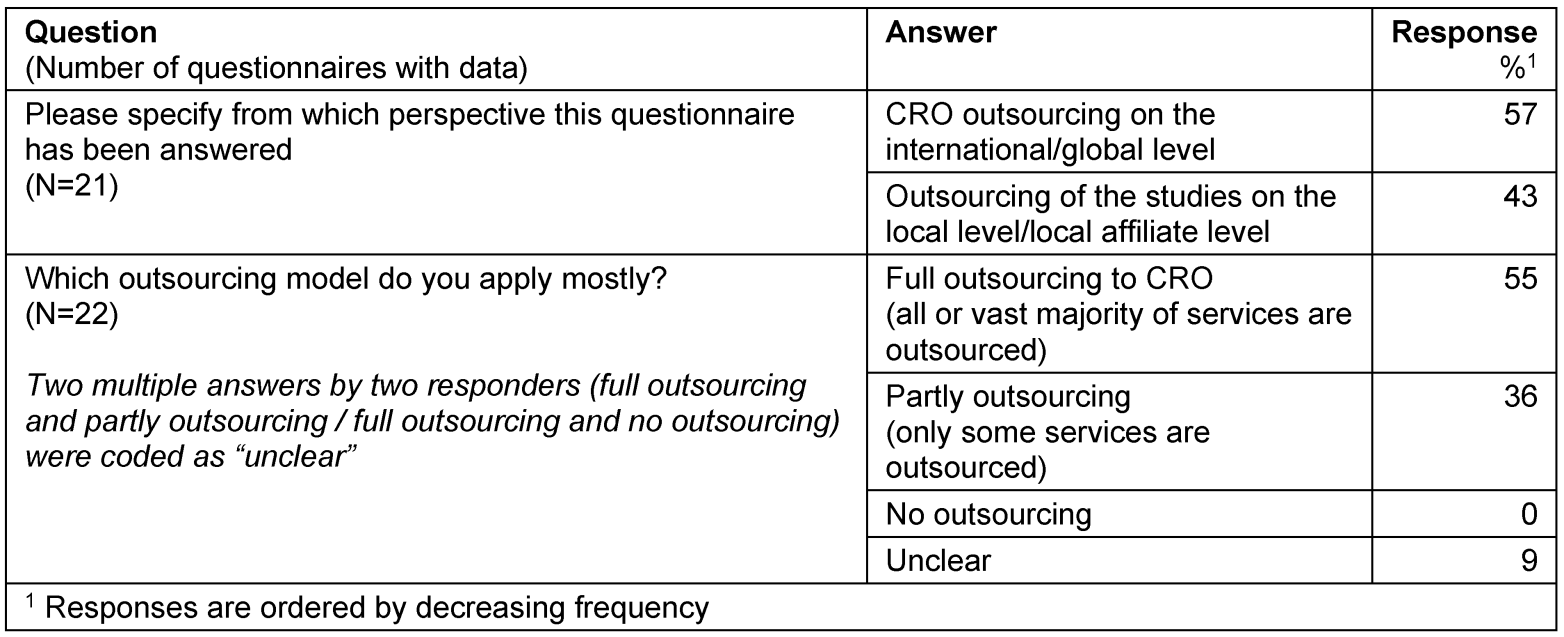
Outsourcing models

**Table 2:**
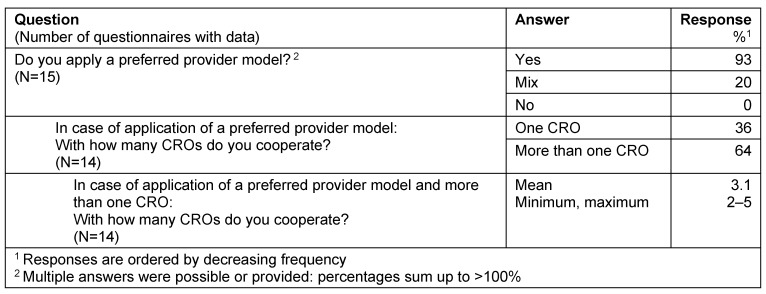
Full outsourcing

**Table 3:**
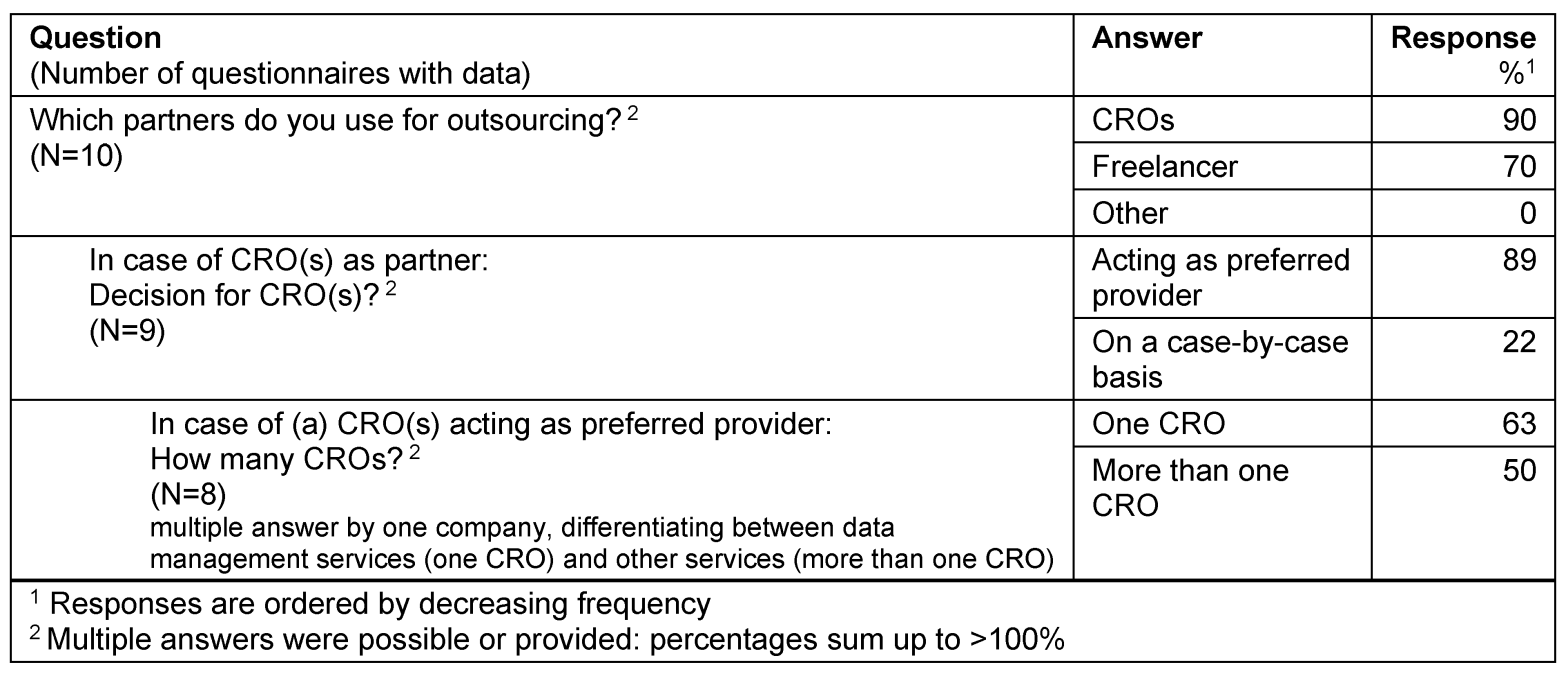
Partly outsourcing

**Table 4:**
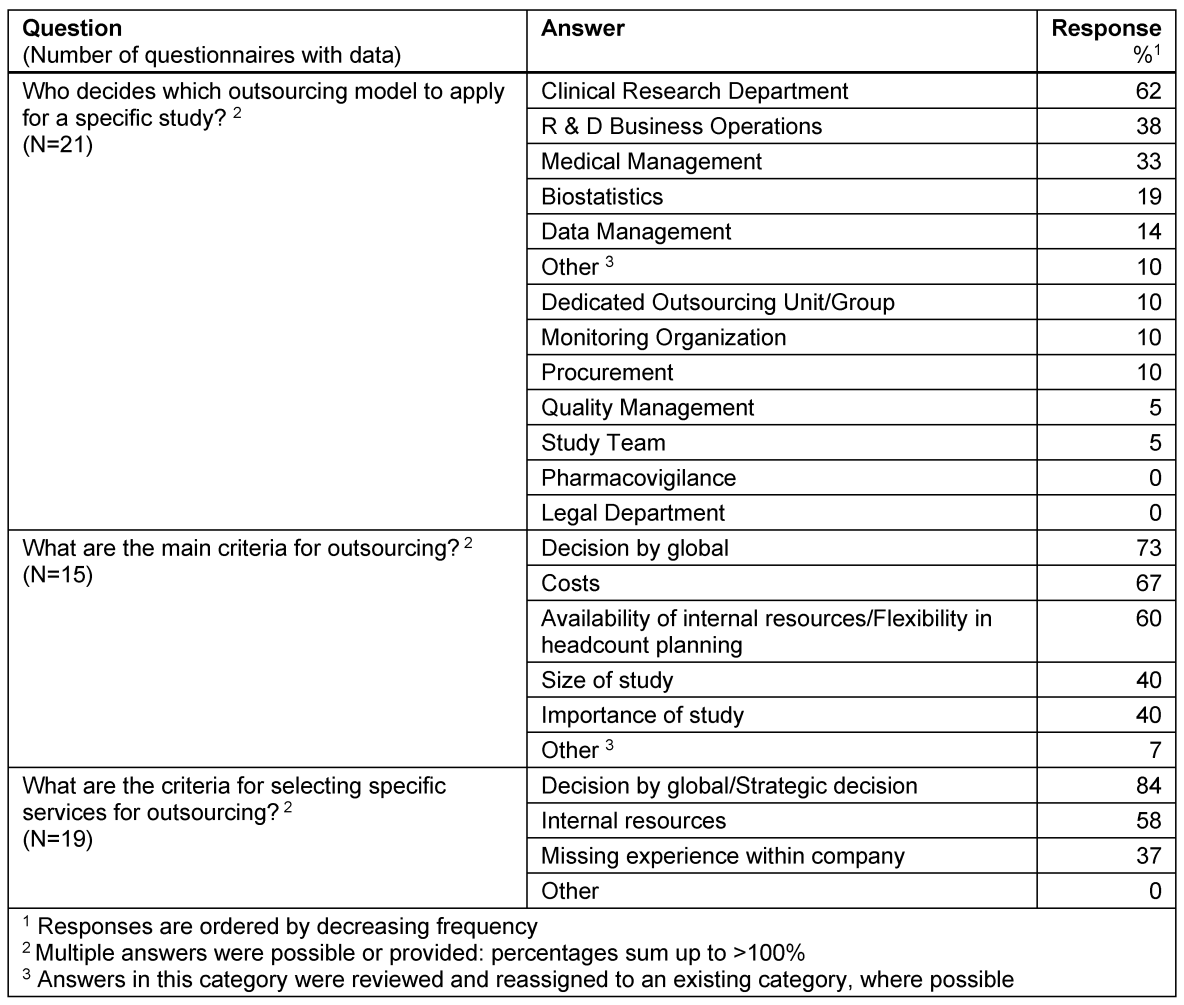
Decision for outsourcing

**Table 5:**
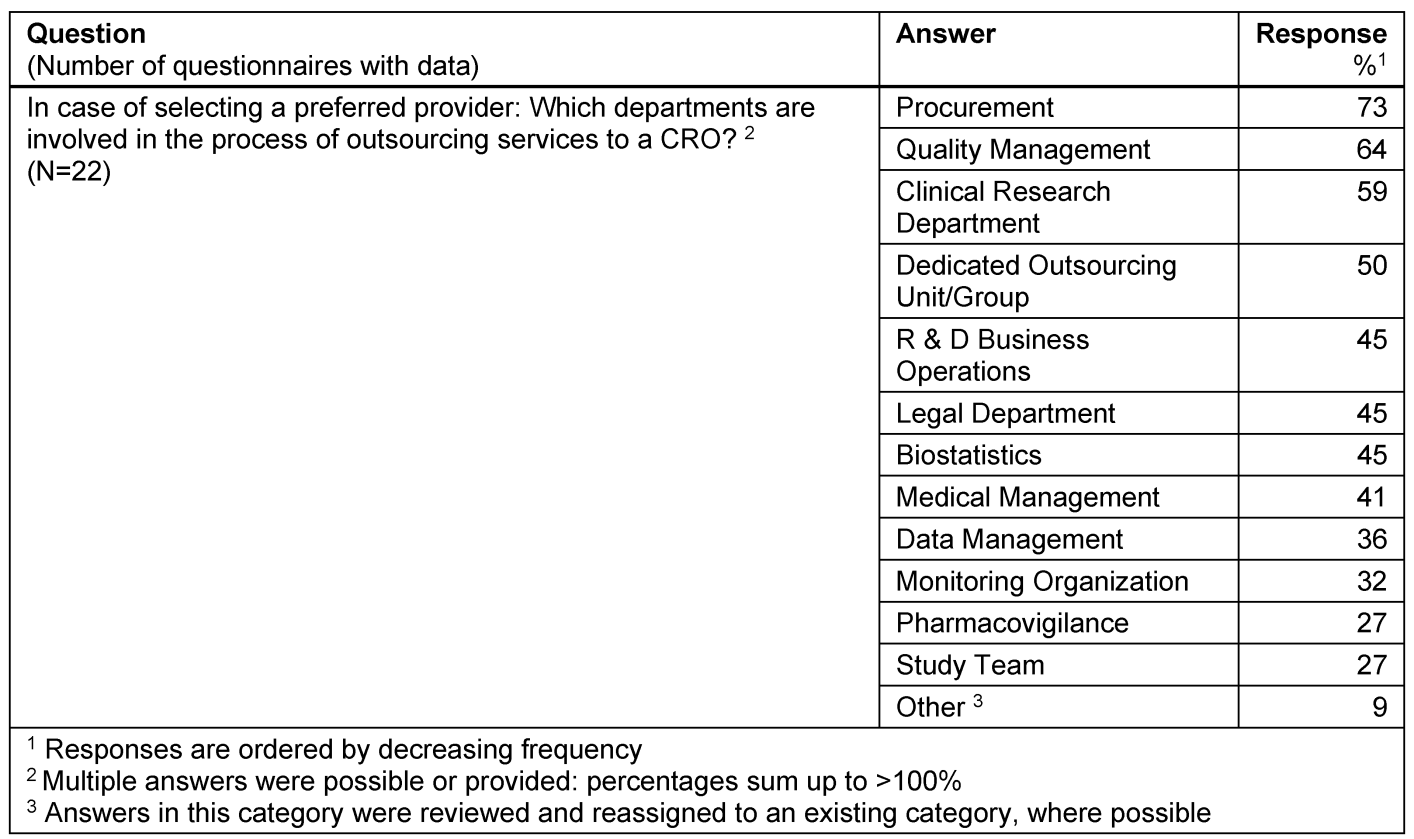
Selection of preferred providers

**Table 6:**
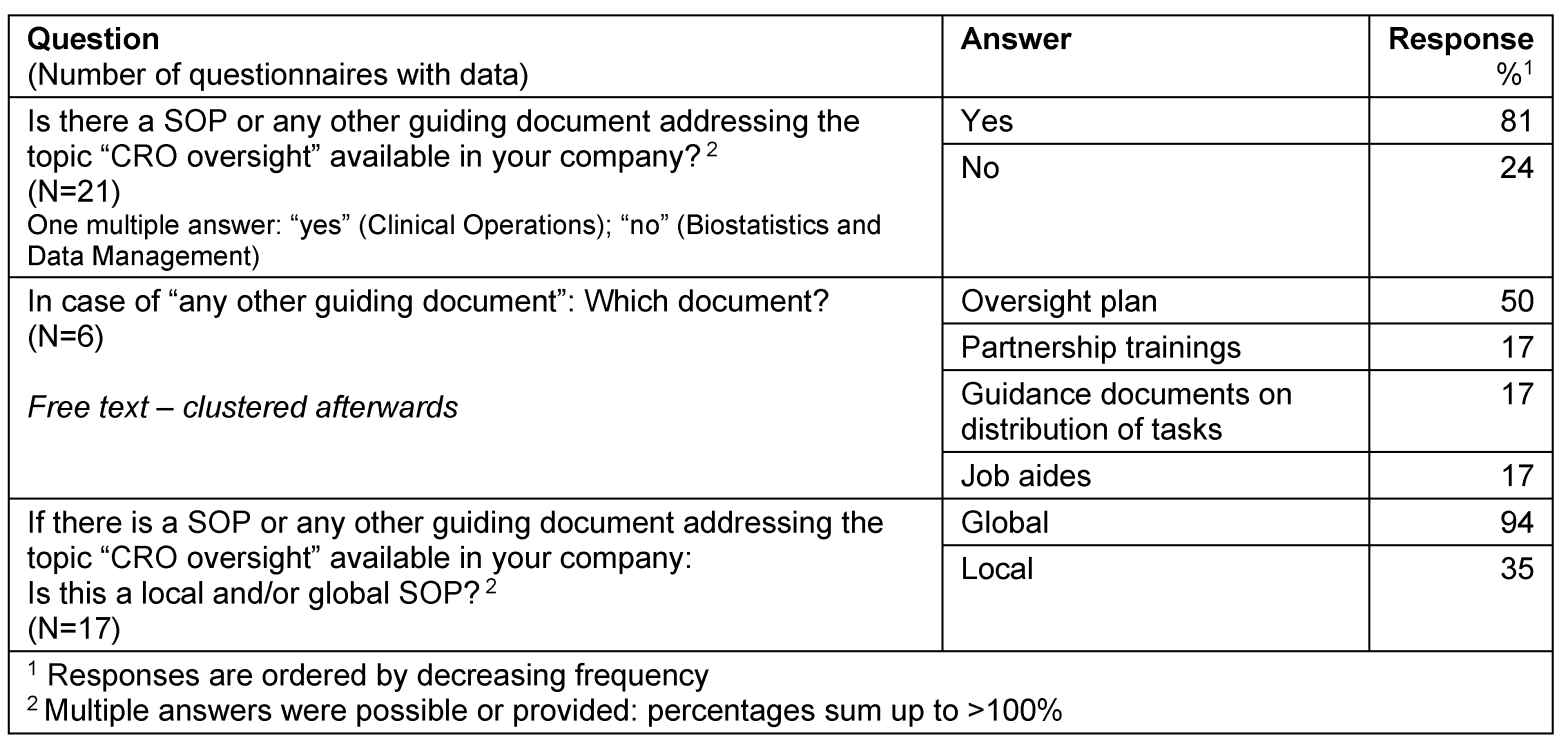
Guiding documents

**Table 7:**
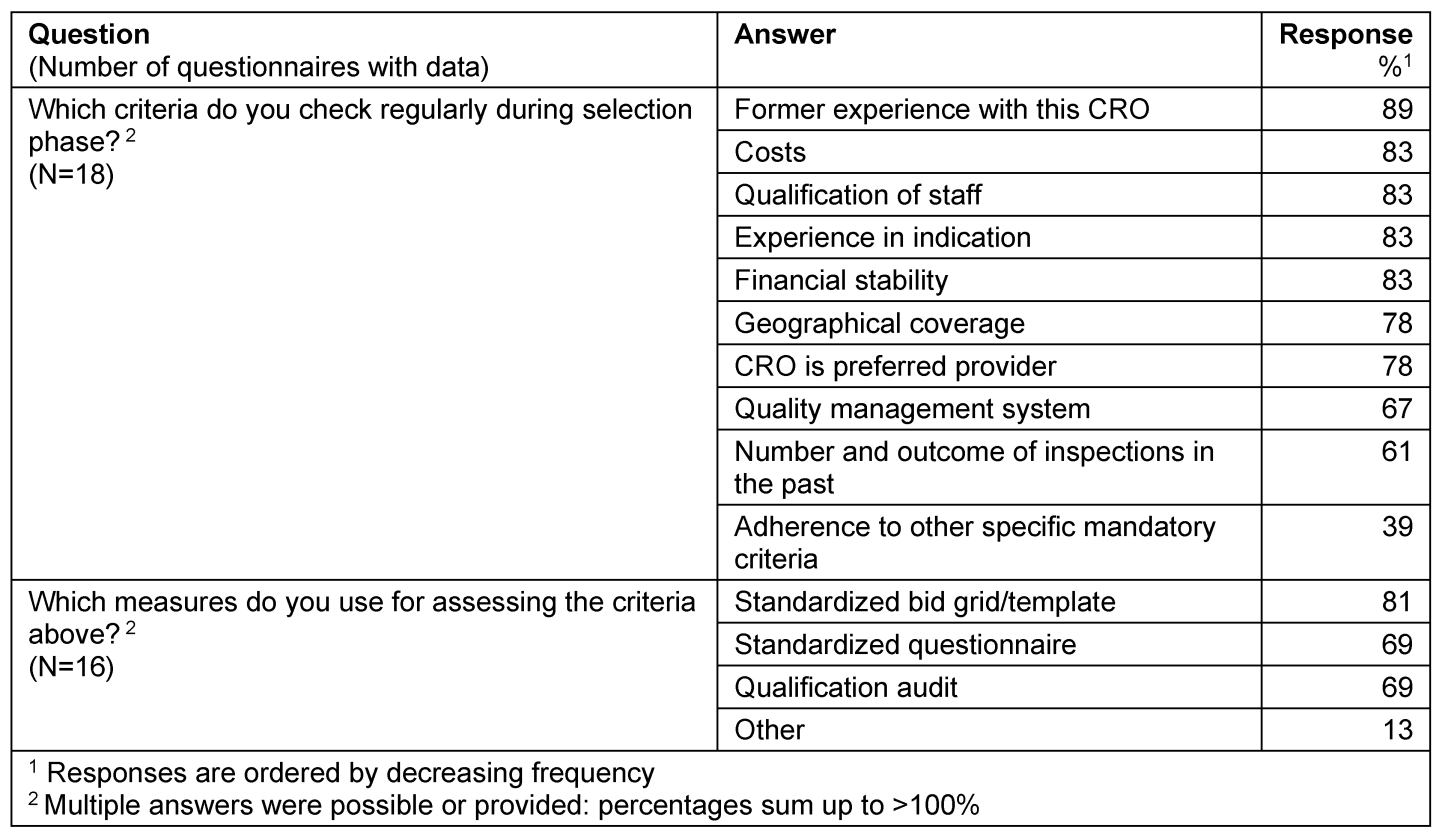
Qualification checks before procurement

**Table 8:**
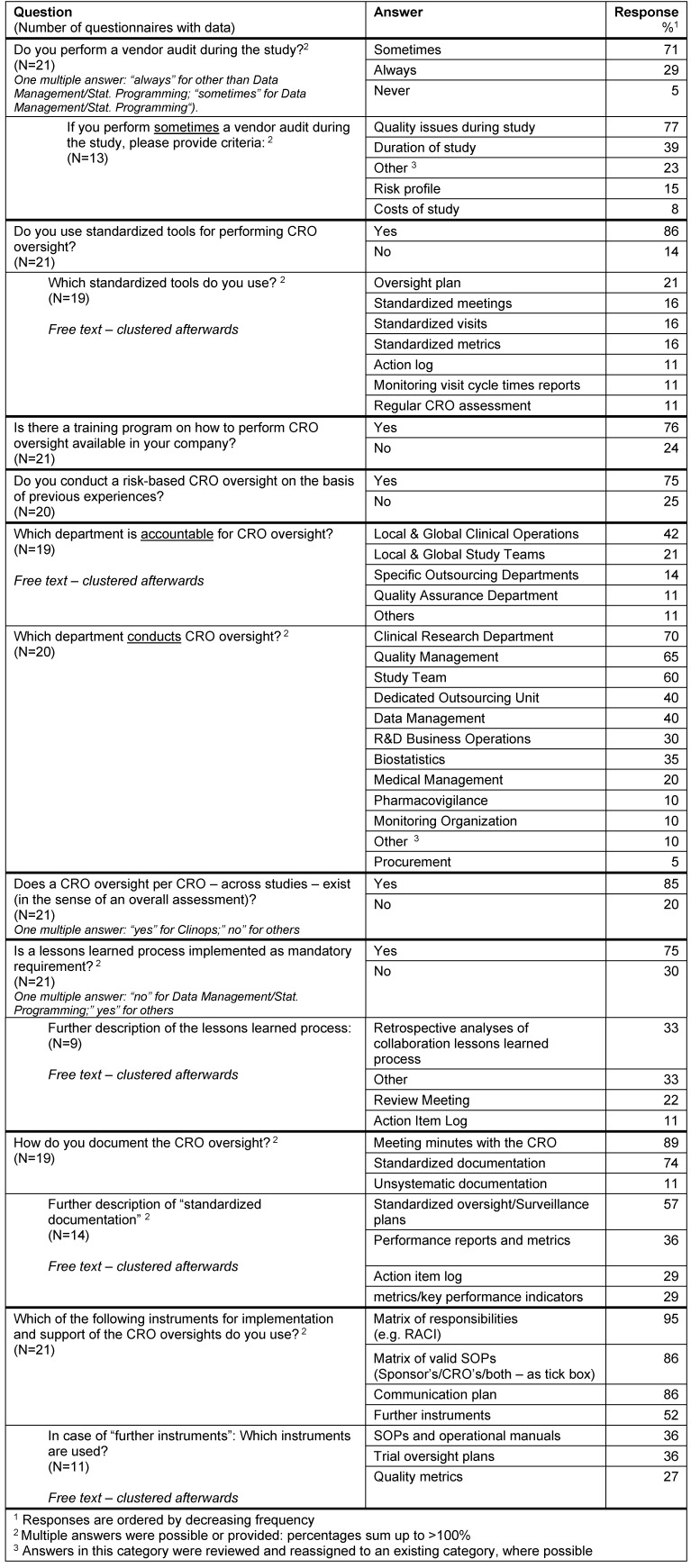
Oversight during study

**Table 9:**
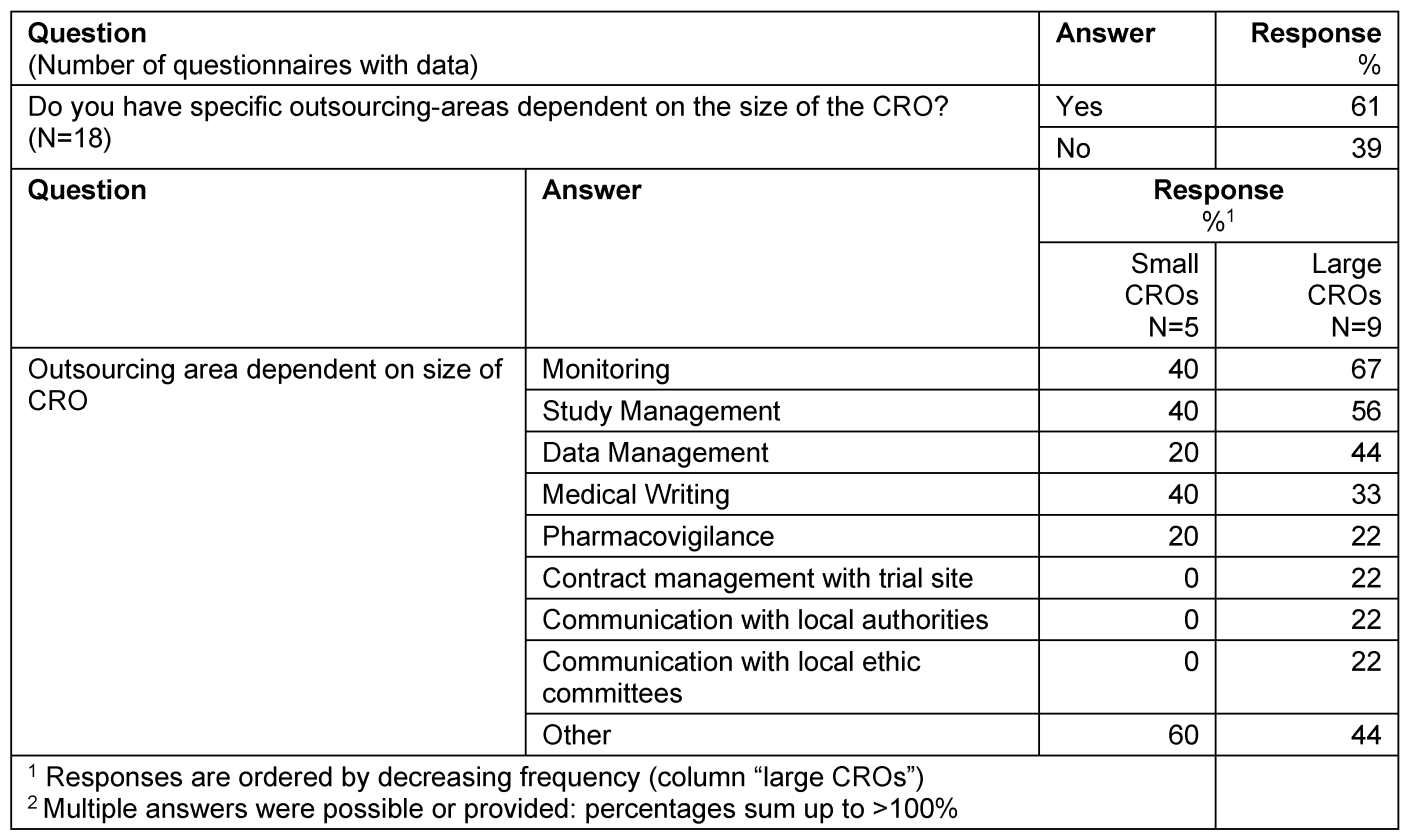
Outsourcing area – size of CRO

**Table 10:**
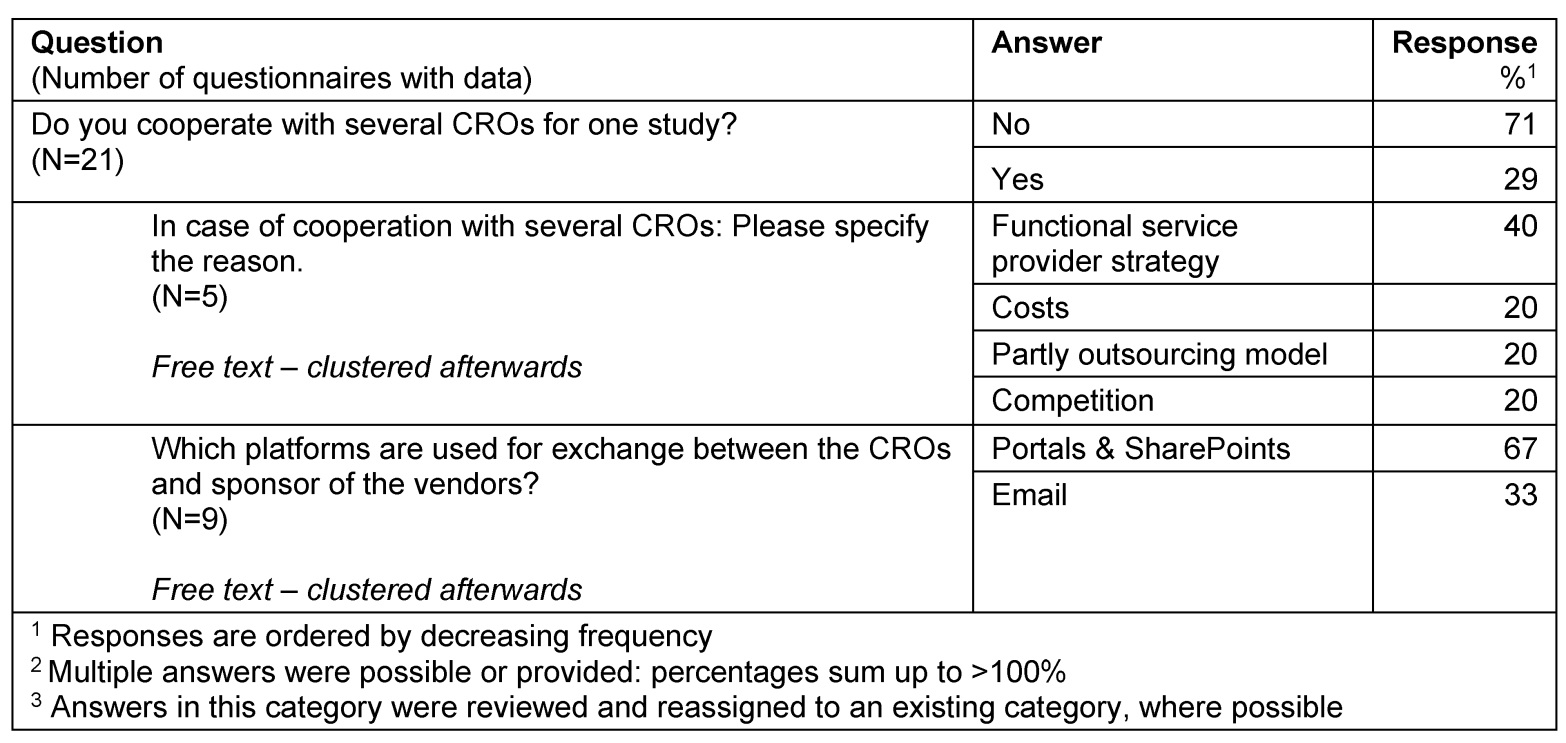
Multiple CROs

**Table 11:**
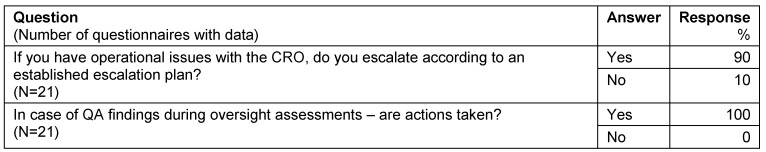
Escalation
