# Supplementary material for: Current practice and perspectives in CRO oversight based on a survey performed among members of the German Association of Research-Based Pharmaceutical Companies (vfa)
Source: Ger Med Sci. 2017 Jan 26;15:Doc02. doi: 10.3205/000243 (PMC5278541; doi:10.3205/000243)
Supplement: Questionnaire on current practice in CRO oversight at vfa companies [file GMS-15-02-s-001.pdf]

Questionnaire on current practice in CRO-Oversight at vfa-companies

Introducing remarks

Content

- This questionnaire refers only to **interventional clinical studies of phases II – IV, local and global**.
- Within this questionnaire the term **“CRO-oversight”** is used for any measure to control the performance, the deliverables and the efficiency of Contract Research Organizations (CROs) performing outsourced tasks on behalf of the pharmaceutical company or acting as the sponsor of a clinical study - not covered in this questionnaire: insourcing/temporary employment. Other terms typically used in this context include “CRO management”, “CRO supervision”.
- Within this questionnaire the term “**preferred provider**” is used for any outsourcing model, in which one or several CROs are selected as primary supplier by a pharmaceutical company in order to perform defined tasks for a series of clinical studies. Other terms typically used in this context include “strategic (alliance) partner/ vendor/CRO”.
- The terms “local” and “global” in regard to this questionnaire refer to international companies with local subsidiaries in various countries. Here “global” refers to the CRO outsourcing on the international level within a company, whereas “local” refers to the German subsidiary and studies on the local German level – if applicable.
- Therefore please make sure that the questionnaire is answered from two different perspectives (global / local) – if applicable. In the case of two different perspectives please provide your answers in two separate questionnaires for the different perspectives.

Name of the Company:

Part 1: General Questions

1. Please specify from which perspective this questionnaire has been answered:

   a. CRO outsourcing on the international/global level

   b. Outsourcing of the studies on the local level/local affiliate level

2. Which Outsourcing-model do you apply mostly?
   a. Full-Outsourcing to CRO (all or vast majority of services are outsourced)
   b. Partly Outsourcing (only some services are outsourced)
   c. No Outsourcing

   Please specify the percentage of the use of the different outsourcing-model:

   (Alternatively: Please specify percentage - sum must be 100%)

3. <u>In case of full-Outsourcing</u>: Do you apply a Preferred Provider model *(at least one answer, in case of Yes or Mixture: one further answer)*

   a. yes
      i. one CRO
      ii. more than one CRO, if yes, how many: ____
   b. Mixture of preferred providers model and single provider
      i. Majority of studies with preferred provider(s)
      ii. Majority of studies without preferred provider(s)
      iii. Equal distribution between single and preferred provider
   c. No

4. <u>In case of **partly** outsourcing (please answer this question only if applicable):</u> Which partners do you use for outsourcing? *(multiple answers)*
   a. CRO(s) *(at least one answer: Yes / No, in case of Yes: at least one further answer)*
      i. yes
         1. acting as Preferred Provider
            a. one CRO
            b. more than one CROs
         2. on a case-by-case basis
      ii. no
   b. Freelancer *(one answer: yes / No)*
      i. yes
      ii. no
   c. Other *(one answer: yes/no)*
      i. yes
         1. Pleas describe further details *(Free Text)*
      ii. No

5. In case of partly outsourcing (please answer this question only if applicable): Which services do you typically outsource? *(multiple answers)*

a. Data Management
b. Biostatistics
c. Medical Writing
d. Monitoring
e. Study Management
f. Pharmacovigilance
g. Communication with ethic committees
h. Communication with competent authorities
i. Medical Review
j. Public Disclosure
k. Other:

Please specify *(Free Text)*

6. Who decides which outsourcing model to apply for a specific study? (*Multiple Answers)*

a. Medical Management
b. Legal Department
c. Procurement
d. Quality Management
e. Biostatistics
f. Data Management
g. Monitoring Organization
h. Clinical Research Department
i. Pharmacovigilance
j. Study Team
k. R & D Business Operations
l. Dedicated Outsourcing Unit / Group
m. Other:

Please specify *(Free Text)*

7. What are the main criteria for outsourcing? (*Multiple Answers, please sort by the importance of the given entry – and label the relevant aspects with numbers from 1 (lowest importance) to 6 (highest importance)*

**Aspect**	**Importance (1-6)**
Availability of internal resources/Flexibility in Headcount planning	
Costs	
Importance of study	
Size of study	
Decision by global	
Other<br>Please specify (Free Text)	

8. What are the criteria for selecting specific services for outsourcing? *(Multiple Answers)*

a. Internal Resources
b. Decision by Global/Strategic decision
c. Missing experience within company
d. Other
   i. Please specify *(Free Text)*

9. Which departments are involved in the process of outsourcing services to a CRO?

1. in case of selecting a preferred provider? *(multiple answers)*

a. Medical Management
b. Legal Department
c. Procurement
d. Quality Management
e. Biostatistics
f. Data Management
g. Monitoring Organization
h. Clinical Research Department
i. Pharmacovigilance
j. Study Team
k. R & D Business Operations
l. Dedicated Outsourcing Unit / Group
m. Other:

Please specify *(Free Text)*

2. in case of outsourcing a study to known preferred provider(s)? *(multiple answers)*
    a. Medical Management
    b. Legal Department
    c. Procurement
    d. Quality Management
    e. Biostatistics
    f. Data Management
    g. Monitoring Organization
    h. Clinical Research Department
    i. Pharmacovigilance
    j. Study Team
    k. R & D Business Operations
    l. Dedicated Outsourcing Unit / Group
    m. Other:

       Please specify *(Free Text)*

3. in case of outsourcing a study and non-existing preferred provider(s)? *(multiple answers)*
    a. Medical Management
    b. Legal Department
    c. Procurement
    d. Quality Management
    e. Biostatistics
    f. Data Management
    g. Monitoring Organization
    h. Clinical Research Department
    i. Pharmacovigilance
    j. Study Team
    k. R & D Business Operations
    l. Dedicated Outsourcing Unit / Group
    m. Other:

       Please specify *(Free Text)*

Part 2 „Oversight“

1. Is there a SOP or any other guiding document addressing the topic “CRO-Oversight“ available in your company?
    a. yes
        i. Is this a local and/or global SOP?
            1. local
            2. global
        ii. Any other documents/guidances – please specify:________
    b. no

2. Check of the CRO`s qualification <u>before</u> procurement:
    a. Which criteria do you check regularly during selection phase? *(multiple answers)*
        i. Financial stability
        ii. Experience in indication
        iii. Former Experience with this CRO
        iv. CRO is preferred provider
        v. Qualification of staff
        vi. Number and outcome of inspections in the past
        vii. Quality Management-system
        viii. Costs
        ix. adherence to other specific mandatory criteria
        x. Geographical coverage
        xi. Other
            1. Please specify *(Free text)*
    b. Which measures do you use for assessing the criteria above?
        i. Qualification-Audit
        ii. Standardized Bid-grid/Template
        iii. Standardized Questionnaire
            1. Please specify standards *(Free text)*
        iv. Other
            1. Please specify (Free Text)

3. Extent and effort of <u>CRO-Oversight during</u> study
   a. Do you perform a vendor audit during the study?
      i. always
      ii. sometimes
         1. Please provide criteria
            a. Duration of study
            b. Costs of study
            c. Quality issues during study
            d. Other
               i. Please specify
      iii. Never

   b. Do you use standardized tools for performing CRO-Oversight?
      i. Yes
         1. Please specify: (e.g. Standardized Metrics, Reports, Meetings, Visits in trial sites)
      ii. no
   c. Is there a training program on how to perform CRO-oversight available in your company?
      i. yes
      ii. no
   d. Do you conduct a risk-based CRO-oversight on the basis of previous experiences?
      i. yes
      ii. no
   e. Which department is accountable for CRO oversight: Please specify….
      i. Medical Management
      ii. Legal Department
      iii. Procurement
      iv. Quality Management
      v. Biostatistics
      vi. Data Management
      vii. Monitoring Organization
      viii. Clinical Research Department
      ix. Pharmacovigilance
      x. Study Team
      xi. R & D Business Operations
      xii. Dedicated Outsourcing Unit / Group
      xiii. Other:
         1. Please specify (Free Text)

f. Which department(s) conducts CRO oversight: Please specify….
    i. Medical Management
    ii. Legal Department
    iii. Procurement
    iv. Quality Management
    v. Biostatistics
    vi. Data Management
    vii. Monitoring Organization
    viii. Clinical Research Department
    ix. Pharmacovigilance
    x. Study Team
    xi. R & D Business Operations
    xii. Dedicated Outsourcing Unit / Group
    xiii. Other:
        1. Please specify (Free Text)

g. Does a CRO-Oversight per CRO - across studies - exist (in the sense of an overall assessment)?
    i. yes
    ii. no

h. Is a Lessons-Learned-Process implemented as mandatory requirement?
    i. yes
        1. Please describe (Free text)
    ii. no

i. How do you document the CRO-Oversight? *(multiple answers)*
    i. Meeting minutes with the CRO
    ii. Standardized documentation (Example: Questionnaires)
    iii. Please specify:
    iv. Unsystematic documentation
    Please specify:

4. Which of the following instruments for implementation and support of the CRO-Oversights do you use? *(multiple answers)*
    a. Matrix of responsibilities (e.g. RACI)
    b. Matrix of valid SOPs (Sponsor's/CRO`s/Both – as tick box)
    c. Communication plan
    d. Further instruments?
        i. Please specify (Free text)

5. Size of CRO

a. Which kind of experience with “small” respectively “large” CROs do you have?

i. Small CRO (purely locally working CROs)

Please sort by the importance of the given entry – and label the relevant aspects with numbers from 1 (lowest importance) to 5 (highest importance)

	Grading
Quality	
Delivery in time	
Costs	
Communication	
Other (please specify – free text)	

ii. Large CRO (multinational CROs)

Please sort by the importance of the given entry – and label the relevant aspects with numbers from 1 (lowest importance) to 5 (highest importance)

	Grading
Quality	
Delivery in time	
Costs	
Communication	
Other (please specify – free text)	

b. Do you have specific outsourcing-areas dependent on the size of the CRO?
    i. yes
        1. Outsourcing area for small CRO

            a. Study Management
            b. Monitoring
            c. Communication with local ethic committees
            d. Communication with local authorities
            e. Contract management with trial site
            f. Data Management
            g. Medical Writing
            h. Pharmacovigilance
            i. Other (Please specify)

        2. Outsourcing area for large CROs

            a. Study Management
            b. Monitoring
            c. Communication with local ethic committees
            d. Communication with local authorities
            e. Contract management with trial site
            f. Data Management
            g. Medical Writing
            h. Pharmacovigilance
            i. Other (Please specify)
    ii. No

6. Do you cooperate with sever*al CROs f*or one study?
    a. Yes
        i. If yes, please specify the reason:_______________________
    b. No

7. Which platforms are used for exchanges between the CROs and sponsor of the Vendors. (Please specify…)

8. If you have operational issues with the CRO, do you escalate according to an established escalation plan?
    a. Yes
        i. Please provide further details on the escalation plan, e.g. which parties are involved *(free text)*
    b. No

9. In case of QA-findings during oversight assessments – are actions taken?
    i. Yes
        1. If yes,:
            a. For which kind of QA-findings (minor, major, critical, observed):_____________
            b. Timeframe until when actions are taken:_________
            c. By which department are actions taken:________
    ii. No

#
